# Supplementary figures and images for: Fomite Transmission Follows Invasion Ecology Principles
Source: mSystems. 2022 May 3;7(3):e00211-22. doi: 10.1128/msystems.00211-22 (PMC9238404; doi:10.1128/msystems.00211-22)

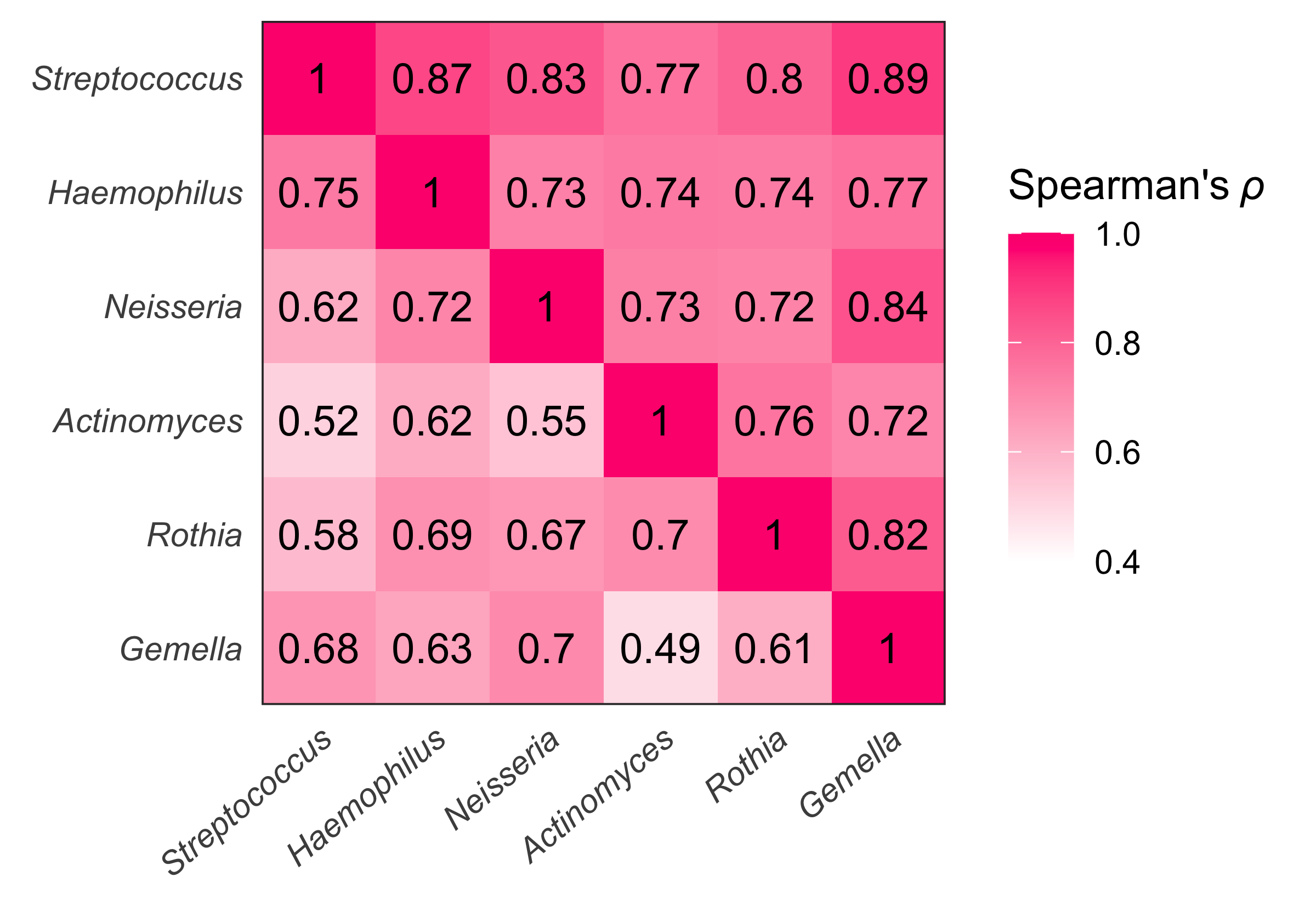

Supplement: FIG S2 [file msystems.00211-22-s0002.tif]
