# Supplementary material for: Fomite Transmission Follows Invasion Ecology Principles
Source: mSystems. 2022 May 3;7(3):e00211-22. doi: 10.1128/msystems.00211-22 (PMC9238404; doi:10.1128/msystems.00211-22)
Supplement: TEXT S1 [file msystems.00211-22-s0009.docx]

**Supplemental texts for methods**

**Amplicon sequence variant inference.** The V3–V4 regions of the 16S rRNA genes of the surface samples were amplified using the primers 341F (5ʹ-CCTAYGGGRBGCASCAG-3ʹ) and 806R (5ʹ-GGACTACNNGGGTATCTAAT-3ʹ), and sequenced on a Novaseq 6000 platform (Illumina, U.S.A.) with PE250 flow cells (Novogene, China). The subsequent demultiplexed paired-end sequence reads with non-biological nucleotides removed were processed using the R package dada2 (v1.16.0) (1), to construct ASVs for microbiota analyses as per the following steps. First, trimming and filtering were performed by trimming the forward and reverse reads at lengths of 227 and 224 bp, respectively, based on their length distribution, whereby the majority of reads were retained and the read lengths were large enough to ensure overlapping for read merging. The shorter and low-quality reads were filtered. Second, read dereplication was performed by combining all identical reads into unique reads with their associated abundances to reduce computational time in the following steps. Third, true reads were inferred based on a parametric error model that first learned the read error rates and then corrected the substitution and indel errors in the reads. Fourth, the reads were merged. The denoised forward and reverse reads were merged with at least a 12-base overlap to obtain contig reads. The unique contigs were the ASVs. ASVs that were much longer (> 439 bp) or shorter (< 403 bp) than expected may be the result of non-specific priming, and they thus were filtered. Fifth, chimeric ASVs were filtered. Chimeric ASVs that could be reconstructed by combining a left segment and a right segment from two more abundant “parent” ASVs were filtered. The abundance of chimeric ASVs accounted for 3.4% of the total ASV abundance. Sixth, taxonomic assignment was performed. A naïve Bayesian classifier method (2) was used to assign taxonomies to the ASVs using the silva_nr99_v138_wSpecies_train_set.fa.gz database (3). Seventh, handoff to R package phyloseq (v1.30.0) (4) was performed. The resulting ASV table, taxonomy table, and associated surface metadata were loaded as a single phyloseq object for downstream analyses.

**Microbiota data quality control.** For the phyloseq object, first, ASVs that did not belong to bacteria or belonged to *Chloroplast* at the order level or *Mitochondria* at the family level were filtered. Second, contaminant ASVs with relative abundances higher than 5% in negative control samples from unoccupied desks were filtered. Third, low-abundance ASVs containing fewer than 10 reads in every sample were filtered to reduce computational time for the downstream analyses. After the quality control steps, 60,247 ASVs were retained. Rarefaction curves demonstrated that the alpha diversities converged at a rarefaction depth of 30,000 reads (Fig. S1), and most of the samples met the sequencing depth threshold of 30,000 reads (Fig. S2). Therefore, the sequencing depth was standardized to a uniform 30,000 reads for downstream analyses.

**Principal coordinates analysis.** PCoA biplots were constructed to reveal microbiota compositional drift through surface touches. The semimetric ecological Bray–Curtis dissimilarities between surfaces were first calculated using the R package vegan (v2.5-6) (5). A square root was then applied to convert the non-Euclidean dissimilarity matrix into a Euclidean matrix. PCoA was then implemented using the R package ape (v5.4-1) (6), to compress the high dimensional dissimilarity matrix and present it as a PCoA biplot.

**Random forest model.** Random forest model was built to identify the indicator ASVs that had statistical indicative powers for hands and private inanimate surfaces. First, a balanced training dataset was assembled using 80% of hands (68 samples) and a similar number of private inanimate surfaces (10 samples each for the phone, mouse, keyboard, desk, cup, chair seatback, and chair arm) from pooled samples from both experiments. Then, the 510 ASVs consistent in the NCM partitions between experiments were selected as feature variables. Next, random forest model was built with 2,000 trees and 20 randomly sampled feature variables as candidates at each split using the R package randomForest (v4.6-14) (7). In the trained random forest model, the importance of each feature variable was ranked based on its statistical indicative powers (i.e., losses in predication accuracy and Gini index). Finally, the accuracy of the trained random forest model was evaluated by the model error rate and the receiver operating characteristic curve using the remaining surfaces.

**Microbiota source tracking.** Microbiota source tracking was conducted to investigate the invader proximity effect at the community level. In the analysis, we assumed that the microbiota on a sink, i.e., a private surface except a hand, belonging to participant P was attributed to four sources: hands of P, hands of P’s “best friend” (identified using STN bipartite projection), hands of the carrier, and the above-neutral microbiota (a synthetic source with the consistent above-neutral ASVs based on NCM partition and the relative abundances averaged from private inanimate surfaces, excluding those belonging to carriers). Only the sinks in which the sources, i.e., P, P’s best friend, and the carrier, were three different individuals were analyzed to avoid duplication.

The source tracking algorithm was implemented using the R package FEAST (v0.1.0) (8), which modeled multiple sources and their corresponding sinks as multinomial distributions. The probability of the observed microbiota compositions can therefore be given as a likelihood function. An expectation-maximization method was then used to identify the local maximum likelihood parameters of the likelihood function, which included the contribution of each source to the sink.

**References**

1. Callahan BJ, McMurdie PJ, Rosen MJ, Han AW, Johnson AJA, Holmes SP. 2016. DADA2: High-resolution sample inference from Illumina amplicon data. Nat Methods 13:581–583.

2. Wang Q, Garrity GM, Tiedje JM, Cole JR. 2007. Naïve bayesian classifier for rapid assignment of rRNA sequences into the new bacterial taxonomy. Appl Environ Microbiol 73:5261.

3. Quast C, Pruesse E, Yilmaz P, Gerken J, Schweer T, Yarza P, Peplies J, Glöckner FO. 2013. The SILVA ribosomal RNA gene database project: Improved data processing and web-based tools. Nucleic Acids Res 41:D590–D596.

4. McMurdie PJ, Holmes S. 2013. phyloseq: An R package for reproducible interactive analysis and graphics of microbiome census data. PLOS One 8:e61217.

5. Oksanen J, Blanchet FG, Kindt R, Legendre P, Minchin P, O’hara R, Simpson G, Solymos P, Stevens M, Wagner H. 2020. vegan: Community ecology package, v2.5–7. <https://CRAN.R-project.org/package=vegan>.

6. Paradis E, Schliep K. 2019. ape 5.0: An environment for modern phylogenetics and evolutionary analyses in R. Bioinformatics 35:526–528.

7. Liaw A, Wiener M. 2002. Classification and regression by randomForest. R News 2:18–22.

8. Shenhav L, Thompson M, Joseph TA, Briscoe L, Furman O, Bogumil D, Mizrahi I, Pe’er I, Halperin E. 2019. FEAST: Fast expectation-maximization for microbial source tracking. Nat Methods 16:627–632.
